# Genetic test to stop smoking (GeTSS) trial protocol: randomised controlled trial of a genetic test (Respiragene) and Auckland formula to assess lung cancer risk

**DOI:** 10.1186/1471-2466-14-77

**Published:** 2014-05-01

**Authors:** John AA Nichols, Paul Grob, Simon de Lusignan, Wendy Kite, Peter Williams

**Affiliations:** 1 Department of Health Care Management and Policy, University of Surrey, Guildford, Surrey GU2 7XH, UK; 2 Smokefree Consultancy Ltd, 3 Peddlars Grove, Yateley, Hants GU46 6AS, UK; 3 Departrment of Mathematics, University of Surrey, Guildford, Surrey GU27XH, UK

**Keywords:** Smoking cessation, Genetic test, Lung cancer

## Abstract

**Background:**

A gene-based estimate of lung cancer risk in smokers has been shown to act as a smoking cessation motivator in hospital recruited subjects. The objective of this trial is to determine if this motivator is as effective in subjects recruited from an NHS primary care unit.

**Method/Design:**

Subjects will be recruited by mailings using smoking entries on the GP electronic data-base (total practice population = 32,048) to identify smokers who may want to quit. Smoking cessation clinics based on medical centre premises will run for eight weeks. Clinics will be randomised to have the gene-based test for estimation of lung cancer risk or to act as controls groups. The primary endpoint will be smoking cessation at eight weeks and six months. Secondary outcomes will include ranking of the gene-based test with other smoking cessation motivators.

**Discussion:**

The results will inform as to whether the gene-based test is both effective as motivator and acceptable to subjects recruited from primary care.

**Trial registration:**

Registered with Clinical Trials.gov, Registration number: NCT01176383.

## Background

Gene testing in primary care is no longer limited by their exorbitant cost. The prices of genetic tests are dropping faster than Moore’s law for computing costs [1]. This leads the focus to shift from cost of genetic testing to the clinical value of individual gene tests. The recent development of gene-based tests that predicts the risk of lung cancer in smokers is an important example [2].

**Figure 1:**
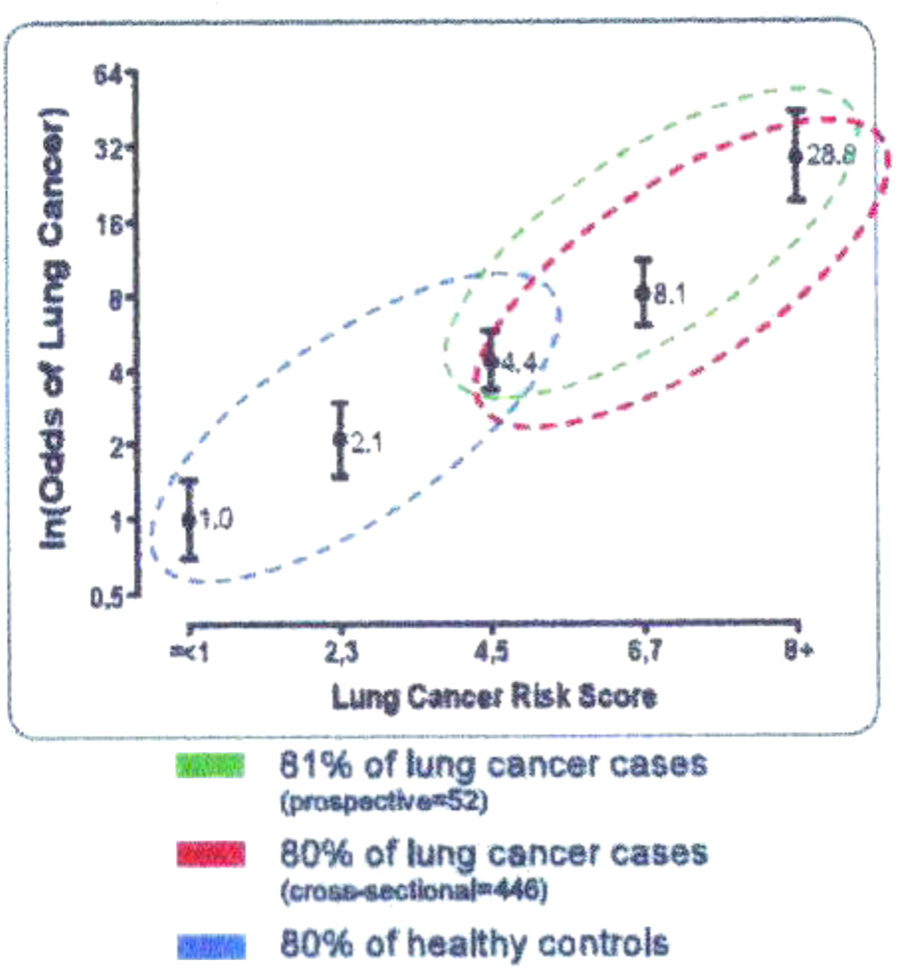
**Research that established the respiragene test.** Distribution of the Respiragene score in a cross-sectional study of 484 control smokers (blue) and 446 with lung cancer (red) (Total = 930) and from the prospective study of 52 lung cancer cases (green). Reference [8].

Despite the well accepted 10-15% probability of lung cancer in smokers, 50% of smokers do not believe they are at significantly increased risk [3]. However, over 80% of smokers would like to know their personal risk of lung cancer [4]. There is a plausible three way link between biomarkers for chronic obstructive pulmonary disease (COPD), a set of 20 single nucleotide polymorphisms (SNPs) associated with cancer risk and lung cancer [5-8] (Figure 1). Research has shown a strong association between a high lung cancer susceptibility score derived from family history of cancer, the 20 SNPs, COPD history (Auckland formula) and the development of lung cancers whereas healthy smokers matched for age, gender and lifetime smoking habits had a relatively low score (n = 446 lung cancer subjects, 484 healthy current smokers). The odds ratio for lung cancer risk varied from 0.2-3.2 depending on the genetic risk (p < 0.001) [9,10]. The accuracy of the Auckland formula in estimating lung cancer risk for a score of >4 was: sensitivity 90%, specificity 45% (Figure 1 which also includes scores for 52 subjects who developed cancer from a six year prospective study of 1212 smokers and ex-smokers). The score for prediction of non-cancer was conducted with a follow up of just six years. It means that 45% of non-cancer subjects have a low cancer score and 55% have some degree of increased score. The 55% with increased scores have simply not been followed up long enough for lung cancers to develop yet. Notwithstanding this limitation there is now a 20 SNP gene test for prediction of lung cancer in smokers under the trade name *Respiragene*.

**Figure 2:**
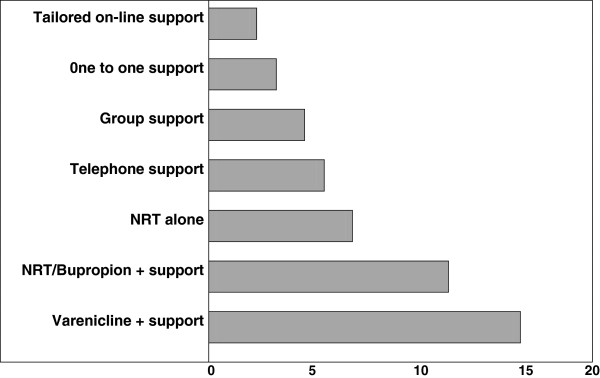
**Efficacy of the variety of smoking cessation strategies.** Percent increase of success for six months over unaided attempts for each type of quitting (chart from West & Shiffman based on Cochrane review data). Totally unaided smoking cessation has a 3-6% success rate. Therefore telephone support (Table 1) increases success rate by 6% = 9-12% quit rate.

Two case-control studies showed a 5-10% increase in cessation with a single gene test of small effect [11,12]. A small smoking cessation pilot study using spirometry results and explanations using the Fletcher-Peto diagram to explain risk demonstrated that patients find this is an acceptable method and the quit rate at 12 months was 27% [13]. In a randomised control trial patients were given either a full explanation of the results of spirometry testing, including an estimation of lung age or just their forced expiratory volume in the first second (FEV1), without explanation (control group). The group of patients who were given the full explanation had a 7.2% higher quit rate than the control group [14]. Data from a hospital outpatient cohort in Auckland suggest a larger increase in quit rate with *Respiragene* test and the Auckland formula (Table 1). Subjects, who were current smokers in the pre-contemplative and contemplative stage, were randomised into either the test group or control group and only the test group had the *Respiragene test*. Counselling and follow-up was done by telephone. Using Auckland formula to incorporate the results of the *Respiragene* test, clinical data and family history a score ranging 1-12 with associated risk level (moderate risk, high risk, very high risk) was calculated and explained to test subjects. Neither group were involved in any formal smoking cessation programme. Indeed, of the 13 subjects that had managed to stop smoking, (28% of the gene-tested group), 48% quit without any medical assistance and only 52% had nicotine replacement therapy [15,16]. When compared with previous studies using telephone counselling alone [17] (Figure 2), there is a 20-25% improvement in smoking cessation with the *Respiragene* test (Table 1). The improvement in intention to quit increases from 56% before testing to 67% in smokers with an average smokers risk of lung cancer or 89% in smokers with a high risk of lung cancer [18].

**Table 1:** Respiragene study in Auckland NZ (n = 43)

	**Cancer susceptibility scale (compared with normal lifetime risk)**	**Cancer susceptibility score**	**Estimated lifetime risk of lung cancer**	**Initial intention to quit**	**Proportion that stopped smoking at 2-4 weeks**	**Proportion still not smoking at 6 months**
Expected result for telephone counselling (Figure 2)	-	-	15%	41%	10-20%	9-12%
Telephone counselling + Respiragene test	1-2.3 (10-35% risk) 2.3-6.7 (35-65% risk) 6.7-8 (65%-80% risk)	27 had average risk score	15%	67%	8/27 (30%)	8 (30%)
		16 had high or very high risk score	30-50% (4-10 times average risk)	89%	10/16 (63%)	6 (37.5%)

Smoking cessation after Respiragene testing and estimation of lung cancer risk with telephone counselling in a small pilot study in Auckland NZ (n = 43) compared with expected quit rate.

A large hospital trial using *Respiragene* for calculating lung cancer susceptibility is currently underway in the USA [19], but there are no planned UK investigations. This study fills that gap and uses the NHS framework for smoking cessation. Other studies have taken place looking at how lung functioning testing in COPD might motivate smokers to quit, suggesting that it is feasible to conduct this sort of study [13,14,20]. This protocol describes a trial to evaluate a gene-based risk test (using genetic and clinical data) as a smoking cessation motivator, in smokers wishing to participate in an NHS primary care smoking cessation clinic (in the action stage of change), alongside the usual counselling and prescribing protocol. It will differ from previous studies using gene testing as a motivator, however, in that the NHS primary care counselling and prescribing protocol will include several other motivators (CO breath testing, saliva cotinine testing and intensive counselling) whereas the Auckland trial, using the same gene test, had none of these. Also, the method of recruitment will differ in that primary care subjects will, of necessity, be different from the Auckland hospital outpatient cohort [15,16].

### Research question

Can the *Respiragene* test combined with an estimation of lung cancer susceptibility be used to increase the uptake, adherence to and success rate in an established smoking cessation programme in subjects who want to quit in a National Health Service, United Kingdom (NHS UK) setting?

### Hypothesis

Genetic testing and estimation of lung cancer susceptibility should increase “smoking cessation outcomes” at six months to >30% (or 1.5-2 fold greater than usual care) irrespective of the risk scores assigned to subjects [11].

## Method/Design

This protocol has been approved by Surrey Research Ethics Committee at the Royal Surrey County Hospital, Guildford, Surrey, UK.

### 1/Recruitment

#### Focus groups

A number of focus groups of different aged smokers will be held to enable them to contribute to the design of the study

### 2/ Recruitment

**Figure 3:**
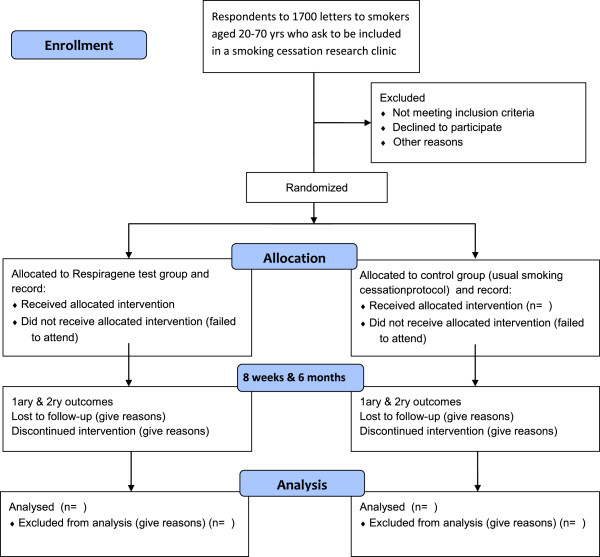
Consort 2010 flow diagram for GeTTS recruitment.

Subjects will be recruited from a large general practice in Surrey (practice population= > 30,000). Smokers aged 20-70 years will be identified from the practice records and contacted by post by their GP. Patients who reply stating that they wish to stop smoking will be randomised (stratified randomisation to ensure equivalent age and gender mix) to two clinics (Figure 3) only one of which will include the gene-based test. Previous trials of genetic testing in association with smoking cessation achieved 83-100% of participants opting for the test depending on the method of recruitment [8,21]. There will be two mailings with SAEs for recruitment with the aim of recruiting at least 30 subjects per clinic (see Power calculations under heading “statistics”). In the first letter the patient’s GP asks the patient to give permission for the researcher to contact him/her to ask about taking part in smoking cessation research (with possible genetic risk testing) and encloses fact sheet 1 and a stamped addressed envelope (SAE) for reply.

Mailing 2. The principal investigator mails patient with Letter 2 to ask him/her if they would like to attend an 8-week smoking cessation clinic and asks if they would be willing to have a test for genetic susceptibility to development of lung cancer and encloses SAE for reply.

Mailing 3. The principal investigator mails Group B subjects and Group A test-concordant subjects to confirm dates of the smoking cessation sessions and full patient information leaflet and consent form enclosed. The information sheet will be slightly different for group A and B. Non-test concordant subjects within group A will be invited to attend the practice nurse for smoking cessation.

### 3/Inclusion and exclusion criteria

i. **Inclusion criteria:** Aged 20-70 years, smoking more than 10 cigarettes daily.

ii. **Exclusion criteria:** Aged under 20 years or over 70 years, smoking less than 10 cigarettes daily, history of major depression and other psychiatric conditions, dementias and serious or terminal illness (cancers etc.). Patients on warfarin would be excluded due to interactions between warfarin and varenicline as varenicline will be used as the modern treatment of choice for smoking cessation. Patients who smoke less than 10 cigarettes/day and patients who did not wish to have a genetic test or do not wish to take part in a research study will be referred to the practice nurse for smoking cessation.

### 4/Smoking cessation clinics

For group A subjects, only subjects who have expressed an interest in having a genetic test and gene-based estimation of susceptibility to lung cancer in mailing 2 will be invited to participate (see referral for decliners above). For group B subjects, all subjects willing to participate are invited to do so. Uptake into smoking cessation programme (i.e. proportion of invitees who accept invitation and attend clinic of those mailed invitation) will be recorded. All subjects who attend the first session of the research clinic will be asked by the principal investigator, JN, to sign a consent form and will be invited to raise any concerns about the protocol (as explained in the full information sheet). The consent form will then be countersigned by JN.

Group A clinics and Group B clinics will be held on different weekdays at the same health centre premises.

Test Subjects who attend Clinic A will be offered a fact sheet on the health risks of smoking (including lung cancer) and the option of the gene-based test for calculation of lung cancer susceptibility whilst subjects who attend Clinic B will be given the same fact sheet on the health risks of smoking (including lung cancer) but without any reference to the gene-based test. The principal investigator will be responsible for handing out the fact sheets and administering the gene-based test in Clinic A and for handing out and explaining the fact sheet in Clinic B.

NHS Surrey’s Smoking Cessation Practitioners will lead in-house smoking cessation clinics A and B using the NHS smoking cessation guidelines [22] under the supervision of the principal investigator at the medical centre. There will be:

•Introductory session which includes a new near patient test for salivary cotinine (nicotine metabolite) – trade name SmokeScreen [23].

•At session 2, patients will be given advice on therapies for smoking cessation. We expect that most patients will opt for a course of varenicline and they will be advised to contact their GP for a prescription.

**Figure 4:**
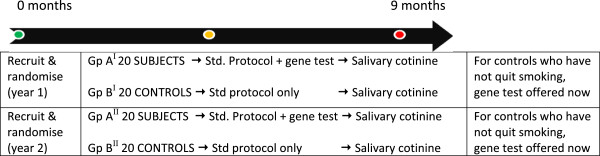
Timeline of project.

**Figure 5:**
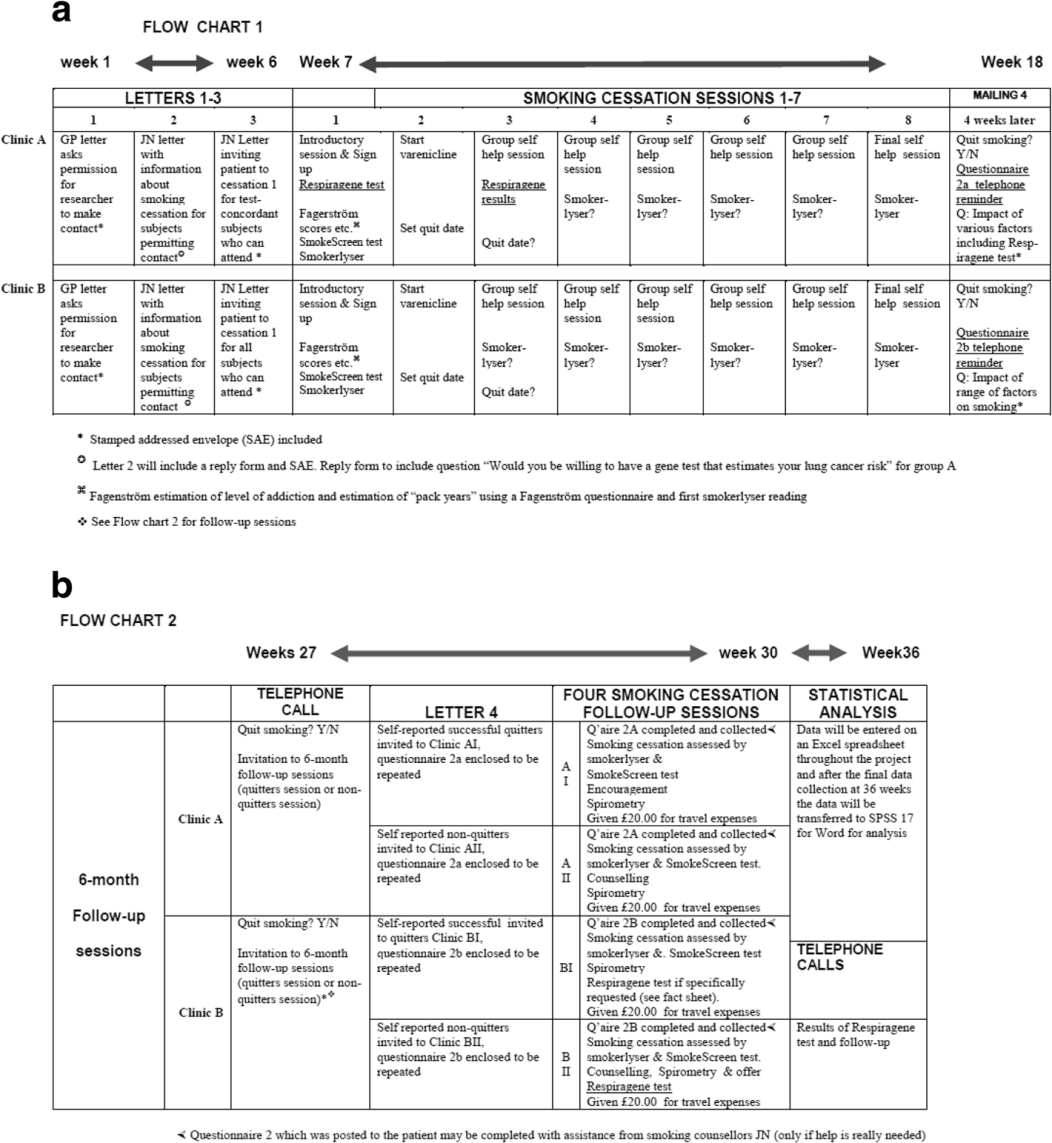
**Flow chart for the duration of the trial. a**. Flow chart of project from start to week 12. **b**. Flow chart of project to week 36.

•This is followed by seven more weekly sessions and a follow-up session at six months (Figure 4). Uptake and adherence to smoking cessation will be monitored by weekly carbon monoxide exhalation measurements (breath test). The principal investigator will be involved in clinic A administering the gene-based test and determining if subjects have COPD from practice records and history in session 1. Participants who are heavy smokers, have a smokers cough and use a salbutamol inhaler can be judged to have COPD even if this is not entered in their GP records (all Group A & B subjects will have spirometry at their 6-month follow-up). Subsequently the principal investigator will report back to clinic A patients with estimated lung cancer risks (session 3). To ensure balance in the control clinic the principal investigator will also attend Clinic B sessions 2 and 3 (see Figure 5 flow charts).

•At the eight week clinic and the 6-month follow-up clinic smoking cessation status and carbon monoxide breath test score will be recorded and a feedback questionnaire used to assess efficacy of various components will be administered.

Mailing 4. Telephone calls followed by letters to patients with invitation to 6-month follow-up session with NHS Smoking Cessation Practitioners and the principal investigator when cessation rate will be assessed and verified by repeating the carbon monoxide breath and salivary cotinine tests.

– for further details see Figure 5: flow charts

We anticipate good attendance at the eight week free smoking cessation clinic, as would be expected if it were a regular NHS smoking cessation clinic but the attendance at the 6-month follow-up clinic may be more challenging. We consider this attendance essential and as attendance will take up an evening of their time, study participants should be paid for their travel expenses (£20) and will receive up to three reminders. Group B subjects attending at the 6-month follow up who have been unable to quit will be offered the gene-based test at this stage.

### Technique for taking the respiragene test

The test requires a Buccal swab and the subjects should not eat or drink within 15 minutes prior to supplying a sample (if has eaten or taken a drink within 15 minutes then rinse mouth with tap water). The nurse taking the sample should wear latex or plastic gloves and take care to avoid contact with the buccal swab collection tip to avoid DNA (deoxyribonucleic acid) contamination. Then:

1. Open buccal swab package at the handle end and carefully remove the swab.

2. Holding handle end of swab stick, scrape the collection tip firmly against the inside of the cheek 5-6 times (about 10 seconds), being careful not to press the plunger that ejects the tip.

3. After taking the sample, eject the swab tip into a labelled 2 ml microcentrifuge tube by firmly pressing the plunger at the end of the handle.

4. Complete and affix the sample tube label onto the microtube. The sample label requires the anonymised trial code for the subject.

### Storage of the respiragene test

After sample collection, tips can be kept at room temperature if they are posted immediately. If storage is necessary, freeze the tubes containing the tips at -20°C.

### Packaging instructions for return of samples to Lab21 Ltd

1. Place absorbent material around the tube and then place tube in the plastic bag provided with the kit. Seal the plastic back as per the instructions on the bag

2. Place the plastic bag containing the sample tube into the shipping box.

3. Seal the box with the security seal supplied.

4. Using the Freepost service provided, send the samples to: Lab 21, 184 Cambridge Science Park, Cambridge CB4 0GA.

Patients will be asked to sign a disclaimer form that explains clearly that this test can only give an estimation of cancer risk and is a test that is still under development (one copy of form for investigators and one for patient).

### Interpretation of result of respiragene test

Lung cancer susceptibility is calculated using the Respiragene test Auckland formula [7]:

Lung cancer score = (number of susceptible genotypes) - (number of protective genotypes) + 3 (for positive family history) + 4 (for past history of COPD) + 4 (for age > 60 years old).

The laboratory reports include the scores with an explanation of how the scores relate to a risk category (see Table 1). When the subject is aged <60 years, the report will also include the score and risk category that would apply if the subject is still a smoker at age 60 years or over.

### Follow-up questionnaires

The questionnaires will be slightly different for groups A (questionnaire 2a) and B (questionnaire 2b) as only 2a will contain a direct reference to the gene-based test. Patients who fail to attend at eight weeks and six months will be contacted by telephone to remind them to complete their questionnaires and hand them in to the practice manager. They are designed to determine which subjects have quit smoking or cut down and which subjects who have failed to quit still plan to do so. There is a section that asks about general motivators and components of the smoking cessation programme. The subjects will be asked to score these motivators and smoking cessation aids for their efficacy in helping them to quit. The questions in this section are almost identical to a validated questionnaire [24]. There are also further questions on whether the subject would recommend the *Respiragene* test to a relative or friend and an open ended question for subjects to add their own comments about the concept of a test that predicts susceptibility to lung cancer in a smoker.

### Data quality assurance

The study has been designed and will be reported in accordance with CONSORT (Consolidated Statement of Reporting Trials) [25]. Data will be controlled in accordance with data protection legislation, institutional protocols of Sussex NHS Research Consortium, and NHS policies for research and information governance for ensuring patient confidentiality [26]. Data will be analysed in SPSS (Statistical Package for Social Sciences) version 15 using an intention to treat approach.

### Outcome measures

#### Primary endpoint

Comparison of smoking cessation rates (7 day point abstinence and continuous abstinence) in Clinic A and Clinic B at 8 weeks and six months.

#### Secondary endpoints

A. Personal data:

1. Number of smokers still smoking who state that they still plan to stop.

2. Daily cigarette consumption of those still smoking.

3. Mean scores for ranking of smoking cessation aids (gene-based test - Clinic A only, salivary cotinine, lung cancer facts - controls in Clinic B only and general counselling from NHS smoking counsellors).

B. Analyse questions about whether subjects would recommend the test to a member of family or a friend.

C. Analyse last (open ended) question using qualitative research methodology.

### Statistics

#### Primary end point

The difference between smoking cessation between Clinic A and Clinic B will be estimated from the four week and six month follow up for the primary endpoint (smoking status confirmed by carbon monoxide breathalyser and salivary cotinine tests). If there is the expected higher rate of smoking cessation for Clinic A compared with Clinic B, statistical significance will be demonstrated by the χ^2^ test.

Since there are, as yet, no case-control studies that compare quit rate following the gene-based test versus quit rate without the test, the expected difference in quit rate between Clinic A and Clinic B is difficult to estimate. Two case-control studies showing only a 5-10% increase in smoking cessation involved just a single gene of small effect [11,12]. In a randomised control trial patients were given either a full explanation of the results of spirometry testing, including an estimation of lung age or just the FEV1, without explanation (control group). The group of patients who were given the full explanation had a 7.2% higher quit rate than the control group. However, data from Auckland suggest a larger uplift of quit rate with *Respiragene.* This can be explained by the superior predictive power of a 20-gene test combined with clinical history (personal history of COPD and family history of lung cancer) to give a rather more impressive estimate of cancer risk than anything previously available.

The adequacy of sample size was tested using data from smoking cessation trials that showed:

•30-40% smoking cessation at 6-months with similar protocols [27,28].

•A 48% quit rate at 2-4 weeks in subjects with high and very high lung cancer risk scores but this difference shrinks to 27% at 6 months.

•Data from Young et al [15,18] (independently verified by McBride et al [11]) that even being given an average score for lung cancer susceptibility increases smoking cessation by approximately 10%.

Therefore, with a minimum sample sizes of 30 per group the following calculations based on these estimated quit rates apply (Table 2). Statistical power of 87.1% is generally acceptable for publication (for alpha error of 5% - i.e. 5% probability of incorrectly rejecting the null hypothesis that there is no difference in the percentage values). For further detailed statistical analysis, refer to Additional file 1.

**Table 2:** Summary of values from which the power of the study are estimated

		**Control group expected quit rate as %ge**	***Respiragene *** **group expected quit rate as %ge**	**χ**^**2 **^**calculated from four-some table**	**P value based on χ**^**2**^	Power calculations*
8 weeks	Sample size 30/30*	70%	94%	5.9	<0.05	79.3%
	Sample size 60/60*			11.7	<0.01	96.9%
6 months	Sample size 30/30**	35%	52%	1.7	NS	36.5%
	Sample size 60/60**			6.2	<0.05	87.1%

*Telephone (alone) quit rate (see Table 1) assumed to be 20%.

**Telephone (alone) quit rate (see Table 1) assumed to be 10-15%.

#### Secondary outcome measures

Similarly, the significance of secondary endpoints on intention to stop smoking, cigarette consumption, uptake of invitation to cessation, adherence to cessation course and self-reported smoking cessation will be calculated by the χ^2^ test but the p value for the ranking scores for information on lung cancer risk and other smoking cessation aids and motivators will be estimated from the unpaired student t-test.

The open ended question: “How do you feel now about having had a genetic test that estimates the probability that you will develop lung cancer at some future date?” will have to be analysed by qualitative analysis to determine the main recurrent themes in responses.

## Discussion

### Overview

Smoking cessation is one of the most cost effective interventions that can be achieved in primary care [29]. However many smokers are very reluctant to commit to a smoking cessation programme (precontemplative and contemplative) and about half of those that attend for smoking cessation intervention (action stage of change) are likely to drop out or give up trying. Therefore, any methodology that increases motivation in both unmotivated and motivated smokers could be very valuable. The gene-based test we are offering has shown promise as a smoking cessation motivator in precontemplative-contemplative smokers in a hospital outpatient setting [15,18] and now needs to be tested out as a motivator for improving adherence in a primary care smoking cessation clinic using a randomised controlled study.

### Strengths

The main strengths of this study are that it is being carried out on subjects from a large primary care population and should therefore be more representative of the general population than previous studies recruited from hospital patients and other special groups. We also have the advantage of being able to carry out this research within the established framework of the local stop smoking service.

### Limitations and assumptions

Although we have estimated, based on previous smoking cessation work using this gene-based test, that the primary endpoint will show that having the test improves quit rate by 20-25%, this was based on a cohort of hospital outpatients in Auckland, New Zealand and subjects recruited from primary care may respond differently. Although we plan to recruit a minimum of 60 subjects, this may not be enough to balance unexpected and unknown confounding factors.

### What we might find

We aim to recruit a minimum of 60 subjects to randomise 30 into group A (test group) and 30 into Group B (control group). The normal experience in NHS smoking cessation clinics is a drop-out rate of 40-50% [30-32]. We need, therefore, to attempt to recruit about 120 subjects in order to get a statistically significant result, based on the assumptions in our power calculations. We may, however, have underestimated the 6-month quit rate using the NHS local stop smoking guidelines [22] which typically involves a multi-interventional programme which includes combinations of varenicline prescriptions, breath carbon monoxide monitoring and intensive counselling giving a quit rate of 70-80% at 6-weeks.There are however no Surrey data for 6-month quit rate which we assume, on the basis of similar smoking cessation data to be about half the 6-week figure [33] ≅ 35%.

An unknown and unpredictable factor, that could skew results significantly, is the possibility that our multi-interventional approach could help to reinforce the health risk message equally for subjects in both groups. Also, the Auckland study design involved recruitment of precontemplative-contemplative smokers from a hospital outpatient setting, compared to this study that will involve primary care subjects who have volunteered to participate in a smoking cessation programme (ie smokers in the action stage of quitting). This population, therefore, could be sufficiently different to give unexpected results. However, the results of this trial will inform as to the acceptability of this approach as well as its effectiveness.

## Abbreviations

CONSORT: Consolidated statement of reporting trials; COPD: Chronic obstructive pulmonary disease; DNA: Deoxyribonucleic acid; NHS: National health service UK; SNP: Single nucleotide polymorphism; SAE: Stamped addresses envelope.

## Competing interests

JN and PG are in receipt of research grants from Lab 21, Cambridge who are marketing the Respiragene test in the UK and Synergenz Bioscience Ltd. who financed the development of the test from its origins in New Zealand. We initially purchased SmokeScreen kits (for salivary cotinine estimation) from GFC Diagnostics Ltd. But they subsequently supplied 30 kits free of charge.

## Authors’ contributions

JN and PG developed the idea of a control trial of the Respiragene test after discussions with Aino Telaranta-Keerie of Lab 21, Cambridge. WK was involved in helping to write the protocol and her experience in running smoking cessation clinics was very helpful. PW was our statistical adviser and SdeL helped us to write the protocol in accordance with CONSORT principles and in development of trial methodology. All authors read and approved the final manuscript.

## Authors’ information

PG is a Visiting Professor of Primary Care at The University of Surrey. SdeL is Professor of Health Care and Clinical Informatics at The University of Surrey. JN is a primary care physician and visiting research fellow at The University of Surrey. WK is a visiting research fellow at The University of Surrey and an experienced smoking cessation nurse. PW is a Statistics Consultant in the Department of Mathematics at The University of Surrey.

## Pre-publication history

The pre-publication history for this paper can be accessed here:

http://www.biomedcentral.com/1471-2466/14/77/prepub

## Supplementary Material

Additional file 1 Detailed statistical analysis. Click here for file

## References

[B1] Wetterstrand KA DNA Sequencing Costs Data from the NHGRI Large-Scale Genome Sequencing Program http://en.wikipedia.org/wiki/Personal_genomics#cite_note-18

[B2] Smerecnik C Grispen JEJ Quaak M Effectiveness of testing for genetic susceptibility to smoking-related diseases on smoking cessation outcomes: a systematic review and meta-analysis Tob Control 2012 21 3 347 354 10.1136/tc.2011.042739 21948804

[B3] Smith SM Campbell MC Macleod U Factors contributing to the time taken to consult with symptoms of lung cancer: a cross sectional study Thorax 2009 64 1953 531 10.1136/thx.2008.096560 19052045

[B4] Sanderson SC O’Neill SC White DB Bepler G Bastian L Lipkus IM McBride CM Responses to online GSTM1 genetic test results among smokers related to patients with lung cancer: a pilot study Cancer Epidemiol Biomarkers Prev 2009 18 7 1953 1961 10.1158/1055-9965.EPI-08-0620 19567511 PMC3417294

[B5] Young RP Hopkins R Black PN Eddy C Wu L Gamble GD Mills GD Garrett JE Eaton TE Rees MI Functional variants of antioxidant genes in smokers with COPD and in those with normal lung function Thorax 2006 61 5 394 399 10.1136/thx.2005.048512 16467073 PMC2111196

[B6] Young RP Hopkins RJ Christmas T Black PN Metcalf P Gamble GD COPD prevalence is increased in lung cancer, independent of age, sex and smoking history Eur Respir J 2009 34 2 380 386 10.1183/09031936.00144208 19196816

[B7] Young RP Hopkins RJ Hay BA Epton MJ Mills GD Black PN Gardner HD Sullivan R Gamble GD Lung cancer susceptibility model based on age, family history and genetic variants PLoS ONE [Electronic Resource] 2009 4 4 e5302 10.1371/journal.pone.0005302 PMC2668761 19390575

[B8] Young RP Hopkins RJ Hay BA Gamble GD GWAS And Candidate SNPs For COPD And Lung Cancer Combine To Identify Lung Cancer Susceptibility: Validation In A Prospective Study Am J Respir Crit Care Med 2010 181 A3738

[B9] Young RP Hopkins RJ Hay BA Epton MJ Mills GD Black PN Gardner HD Sullivan R Gamble GD A gene-based risk score for lung cancer susceptibility in smokers and ex-smokers Postgrad Med J 2009 85 515 524 10.1136/pgmj.2008.077107 19789190

[B10] Young RP Hopkins RJ Hay BA Epton MJ Black PN Gamble GD Lung cancer gene associated with COPD: triple whammy or possible confounding effect? Eur Respir J 2008 32 5 1158 1164 10.1183/09031936.00093908 18978134

[B11] McBride CM Bepler G Lipkus IM Lyna P Samsa G Albright J Datta S Rimer BK Incorporating genetic susceptibility feedback into a smoking cessation program for African-American smokers with low income Cancer Epidemiol Biomarkers Prev 2002 11 6 521 528 12050092

[B12] Sanderson SC Humphries SE Hubbart C Hughes E Jarvis MJ Wardle J Psychological and Behavioural Impact of Genetic Testing Smokers for Lung Cancer Risk: A Phase II Exploratory Trial J Health Psychol 2008 13 481 494 10.1177/1359105308088519 18420756

[B13] Wells S de Lusignan S Does screening for loss of lung function help smokers give up? Br J Nurs 2003 12 12 744 750 12829957 10.12968/bjon.2003.12.12.11337

[B14] Parkes G Greenhalgh T Griffin M Dent R Effect on smoking quit rate of telling patients their lung age: The Step2quit randomised control trial BMJ 2008 336 598 600 10.1136/bmj.39503.582396.25 18326503 PMC2267989

[B15] Hopkins RJ Young RP Hay B Gamble GD Lung cancer risk testing enhances NRT uptake and quit rates in randomly recruited smokers offered a gene based risk test Am J Respir Crit Care Med 2012 185 A2590

[B16] Hopkins RJ Young RP Hay B Gamble GD Gene-based lung cancer risk score triggers smoking cessation in randomly recruited smokers Am J Respir Crit Care Med 2011 183 A5441

[B17] West R Shiffman S McLean D Fast Facts: Smoking Cessation (Fast Facts series) – Paperback 2007 London: Health Press

[B18] Young RP Hopkins RJ Smith M Hogarth DK Smoking cessation: the potential role of risk assessment tools as motivational triggers [Review] Postgrad Med J 2010 86 1011 26 33 10.1136/pgmj.2009.084947 20065338

[B19] Cabebe E Recruitment details: REACT Clinical Trial Lung Cancer Detection Study http://www.elcaminohospital.org/Cancer_Center/Clinical_Trials/Lung_Cancer_Detection_Study

[B20] McClure JB Ludman EJ Grothaus L Pabiniak C Richards J Impact of spirometry feedback and brief motivational counseling on long-term smoking outcomes: a comparison of smokers with and without lung impairment Patient Education & Counseling 2010 80 2 280 283 10.1016/j.pec.2009.11.002 20434863 PMC2897973

[B21] Sanderson SK Humphries SE Hubbart C Psychological and behavioural impact of genetic testing smokers for lung cancer risk J Health Psychol 2010 13 4 481 494 18420756 10.1177/1359105308088519

[B22] Croghan E NHS Local stop smoking services, services delivery and monitoring guidance 2011/12 https://www.gov.uk/government/uploads/system/uploads/attachment_data/file/213755/dh_125939.pdf

[B23] Barnfather KD Cope GF Chapple IL Effect of incorporating a 10 minute point of care test for salivary nicotine metabolites into a general practice based smoking cessation programme: randomised controlled trial BMJ 2005 331 999 10.1136/bmj.38621.463900.7C 16210250 PMC1273454

[B24] West R Smoking toolkit study: protocol and methods 2006 http://www.smokinginengland.info/sts-documents/ (ST5001)

[B25] Moher D Hopewell S Schulz KF Montori V Gotzsche PC Devereaux PJ Elbourne D Egger M Altman DG CONSORT 2010 explanation and elaboration: updated guidelines for reporting parallel group randomised trials BMJ 2010 340 c869 10.1136/bmj.c869 20332511 PMC2844943

[B26] DH/IPU/Patient Confidentiality Department of Health: Confidentiality – NHS Code of practice 2003 http://webarchive.nationalarchives.gov.uk/20130107105354/http://www.dh.gov.uk/prod_consum_dh/groups/dh_digitalassets/@dh/@en/documents/digitalasset/dh_4069254.pdf

[B27] Lee JH Jones PG Bybee K O’Keefe JH A longer course of varenicline therapy improves smoking cessation rates [Review] Prev Cardiol 2008 11 4 210 214 10.1111/j.1751-7141.2008.00003.x 19476573

[B28] West R Sohal T “Catastrophic” pathways to smoking cessation: findings from national survey BMJ 2006 332 458 460 10.1136/bmj.38723.573866.AE 16443610 PMC1382540

[B29] Kahenda JW Loomis BR Anhikara B Marshall L A review of economic evaluations of tobacco control programs Int J Environ Res Public Health 2009 6 1 51 68 19440269 10.3390/ijerph6010051 PMC2672319

[B30] Sanders D Fowler G Mant D Fuller A Jones L Marzillier J Randomized controlled trial of anti-smoking advice by nurses in general practice J R Coll Gen Pract 1989 39 324 273 276 2556540 PMC1711904

[B31] Wennike P Danielsson T Björn B Westin A Tøneson P Smoking reduction promotes smoking cessation: results from a double blind, randomized, placebo-controlled trial of nicotine gum with 2-year follow-up Addiction 2003 98 10 1395 1402 10.1046/j.1360-0443.2003.00489.x 14519176

[B32] MacPherson L Stipelman BA Duplinsky M Lejuez CW Distress tolerance and pre-smoking treatment attrition: examination of moderating relationships Addict Behaviour 2008 33 11 1385 1393 10.1016/j.addbeh.2008.07.001 PMC2561069 18706768

[B33] Soulier-Parmeggiani L Griscom S Bongard O Avvanzino R Bounameaux H One-year results of a smoking-cessation programme Journal Suisse de Médecine 1999 129 10 395 398 10212973

