# Supplementary material for: Genetic test to stop smoking (GeTSS) trial protocol: randomised controlled trial of a genetic test (Respiragene) and Auckland formula to assess lung cancer risk
Source: BMC Pulm Med. 2014 May 1;14:77. doi: 10.1186/1471-2466-14-77 (PMC4108019; doi:10.1186/1471-2466-14-77)
Supplement: Additional file 1 — Detailed statistical analysis. [file 1471-2466-14-77-S1.doc]

**Additional file 1**. **Detailed statistical analysis**

For 8-week quit rate (attendance at the last of eight smoking cessation clinic sessions), assuming:

- Clinic size of 30 subjects per clinic (30/30)
- In Young’s small pilot study and assuming telephone (only) quit rate of 20%, then uplift of quit rate for *Respiragene* test & estimation of cancer risk is:
  - 30%-20% = 10% for average risk score
  - 63%-20% = 38% for high and very high risk score
- That for subjects in Clinic A who have *Respiragene* test & estimation of cancer risk, 10-15 will have average risk scores and 10-15 will have high or very high risk scores
- That normal quit rate using our varenicline + counselling protocol is at least 70% at the 8-week clinic attendance and non-quit rate is 30% = 21 quitters and 9 non-quitters
- 50% with high and very high cancer risk scores will have an uplift in quit rate of 30% and 50% with average cancer risk scores will have an uplift of quit rate of 63%
- Then in clinic A (the test group clinic) assuming a 15/15 split of high/average scores:
  - Of 15 subjects with average cancer risk scores 10% more subjects will quit = 10% of 15 = 1.5
  - Of 15 subjects with high & very high risk score 48% more subjects will quit = 38% of 15 = 5.7
  -  quit rate = 21+1.5+5.7 = 28.2

Therefore the test group (Clinic A) will have 28.2 quitters and 1.8 non-quitters

Control clinic (Clinic B) will have 21 quitters and 9 non-quitters

|  | Quitters | Non-quitters |  |
| --- | --- | --- | --- |
| Clinic A | a | b | a+b = 30 |
| (test group) | 28.2 | 1.8 |  |
| Clinic B | c | d | c+d = 30 |
| (control group) | 21 | 9 |  |
|  | a+c = 49.2 | b+d = 10.8 | a+b+c+d = 60 |
|  | a x d = 253.8 | |  |
|  | b x c = 37.8 | |  |
|  | ad-bc = 216 | |  |
| χ^2^ = (bc – ad)^2^ x (a+b+c+d) ÷ (a+b x c+d x a+c x b+d) | | | |
| = 5.85 | | | |

χ^2^ of 5.85 with two degrees of freedom p<0.05 with Power of 79.3% for Alpha Error of 5%

**Estimated six month quit data**

For six month quit rate, assuming:

- a clinic size of 30 subjects per clinic (30/30)
- In J Young’s small pilot study and assuming telephone (only) mean quit rate of 15%, then uplift of quit rate for *Respiragene* test & estimation of cancer risk is:
  - 30%-15% = 15% for average risk score
  - 33%-15% = 18% for high and very high risk score
- That for subjects in Clinic A who have *Respiragene* test & estimation of cancer risk, 10-15 will have average risk scores and 10-15 will have high or very high risk scores
- That normal quit rate using our varenicline + counselling protocol is at least 70% at 4 weeks and non-quit rate is 30% = 21 quitters (and 9 non-quitters) but is likely to halve at 6-month follow-up =10.5 quitters (and 19.5 non-quitters)
- 50% with high and very high cancer risk scores will have an uplift in quit rate of 18% and 50% with average cancer risk scores will have an uplift of quit rate of 15%
- Then, in clinic A (the test group clinic) assuming a 15/15 split of high/low scores:
  - Of 15 subjects with average cancer risk scores 15% more subjects will quit = 15% of 15 = 2.25
  - Of 15 subjects with high & very high risk score 48% more subjects will quit = 18% of 15 = 2.7
  -  quit rate = 10.5+2.25+2.7 = 15.5

Therefore Clinic A (test group) will have 15.5 quitters and 14.5 non-quitters

Clinic B (control group) will have 10.5 quitters and 19.5 non-quitters

|  | Quitters | Non-quitters |  |
| --- | --- | --- | --- |
| Clinic A | a | b | a+b = 30 |
| (test group) | 15.5 | 14.5 |  |
| Clinic B | c | d | c+d = 30 |
| (control group) | 10.5 | 19.5 |  |
|  | a+c = 26 | b+d = 34 | a+b+c+d = 60 |
|  | a x d = 302.25 | |  |
|  | b x c = 152.25 | |  |
|  | ad - bc = 216 | |  |
| χ^2^ = (bc – ad)^2^ x (a+b+c+d) ÷ (a+b x c+d x a+c x b+d) | | | |
| = 1.7 | | | |

χ^2^ = 1.7, statistically NS (but this data does not take account of the effect of the reinforcement of the lung cancer risk scores for Clinic A subjects that would take place in a 8-week smoking cessation course)

Statistical power = 36.5% for alpha error 5%

Possible sources of error are:

- Auckland data may be specific to the New Zealand response to lung cancer risk and subjects in semi-rural Surrey may respond differently to estimation of lung cancer risk
- Confounding factors in comparison of the two clinics (age/gender mix, social class, mean level of nicotine addiction etc.)
- Underestimation of 6-month quit rate using Surrey PCT smoking cessation protocol which typically includes combinations of varenicline with intensive counselling giving a quit rate of 70-80% at 6-weeks – there are however no Surrey data for 6-month quit rate which we assume, on the basis of similar smoking cessation data to be about half the 6-week figure [32] ≅ 35%.

Similar calculations were carried out on the basis of recruiting 120 subjects to give 60 test subjects and 60 controls (Table 2) although we are unlikely to achieve this level of recruitment.
